# Genome-wide identification and expression pattern analysis of the kiwifruit GRAS transcription factor family in response to salt stress

**DOI:** 10.1186/s12864-023-09915-z

**Published:** 2024-01-02

**Authors:** Ling Zhu, Tuo Yin, Mengjie Zhang, Xiuyao Yang, Jiexin Wu, Hanbing Cai, Na Yang, Xulin Li, Ke Wen, Daming Chen, Hanyao Zhang, Xiaozhen Liu

**Affiliations:** 1 Key Laboratory for Forest Resources Conservation and Utilization in the Southwest Mountains of China, Ministry of Education, Southwest Forestry University, Kunming, 650224 China; 2 Key Laboratory of Biodiversity Conservation in Southwest China, National Forest and Grassland Administration, Southwest Forestry University, Kunming, 650224 Yunnan Province China; 3 Research Institute of Agriculture Ecological in Hot Areas, Yunnan Academy of Agricultural Science, Yuan Mou, Yunnan, 651300 China

**Keywords:** Kiwifruit, GRAS transcription factors, Genome-wide analysis, Transcriptome, Salt stress, Expression patterns

## Abstract

**Background:**

GRAS is a family of plant-specific transcription factors (TFs) that play a vital role in plant growth and development and response to adversity stress. However, systematic studies of the GRAS TF family in kiwifruit have not been reported.

**Results:**

In this study, we used a bioinformatics approach to identify eighty-six AcGRAS TFs located on twenty-six chromosomes and phylogenetic analysis classified them into ten subfamilies. It was found that the gene structure is relatively conserved for these genes and that fragmental duplication is the prime force for the evolution of *AcGRAS* genes. However, the promoter region of the *AcGRAS* genes mainly contains cis-acting elements related to hormones and environmental stresses, similar to the results of GO and KEGG enrichment analysis, suggesting that hormone signaling pathways of the AcGRAS family play a vital role in regulating plant growth and development and adversity stress. Protein interaction network analysis showed that the AcGRAS51 protein is a relational protein linking DELLA, SCR, and SHR subfamily proteins. The results demonstrated that 81 genes were expressed in kiwifruit *AcGRAS* under salt stress, including 17 differentially expressed genes, 13 upregulated, and four downregulated. This indicates that the upregulated *AcGRAS55, AcGRAS69*, *AcGRAS86* and other *GRAS* genes can reduce the salt damage caused by kiwifruit plants by positively regulating salt stress, thus improving the salt tolerance of the plants.

**Conclusions:**

These results provide a theoretical basis for future exploration of the characteristics and functions of more *AcGRAS* genes. This study provides a basis for further research on kiwifruit breeding for resistance to salt stress. RT-qPCR analysis showed that the expression of 3 *AcGRAS* genes was elevated under salt stress, indicating that *AcGRAS* exhibited a specific expression pattern under salt stress conditions.

**Supplementary Information:**

The online version contains supplementary material available at 10.1186/s12864-023-09915-z.

## Background

Kiwifruit is a deciduous perennial vine of the *Actinidia* genus in the family Actinidiaceae [1]. Its fruit contains a large amount of organic matter, essential minerals, and vitamins, especially vitamin C, which is much higher than that of oranges, apples, and pears, making it the "king of fruits" and " king of vitamin C " with a high nutritional value [2]. According to FAO data in 2019, the total production of kiwifruit in the world was 4.34 million tons, of which approximately 2.2 million tons were in China, mainly *Actinidia chinensis* and *A. deliciosa* [3]. In contrast, the statistical analysis of the Chinese Horticultural Society Kiwifruit Branch in 2020 showed that the actual planted area of kiwifruit in China has exceeded 290,000 hm2, and the planted area of *A. deliciosa* and *A. chinensis* is relatively high [4]. However, the increasing salinization of the soil has greatly impacted kiwifruit production. Some studies have shown that the control of salinized soils is mainly achieved through the selection and breeding of salt-tolerant varieties, cross-breeding, mutagenesis, and transgenic and cellular engineering [5]. Genetic engineering techniques are considered one of the most reliable and effective methods [6]. *A. chinensis* var. *deliciosa* cv. Xuxiang is one of the most salt-resistant of many kiwifruit varieties [7], and it is vital to investigate its salt-resistance-related genes and salt-resistance mechanism.

Transcription factors (TFs) are binding proteins that interact specifically with cis-acting elements. If they interact with each other and with other related proteins, they can regulate the expression level of gene transcription by repressing or activating the transcription process [8]. In recent years, with the rapid development of high-throughput sequencing technology, whole-genome sequencing has been completed in most plants, such as *Arabidopsis thaliana* [9], *Oryza sativa* [10], *Glycine max* [11] and *Gossypium hirsutum* [12]. A series of TFs that regulate the expression of genes related to drought, high salinity, low temperature, and development have been successively isolated from many plants [8], such as SAP2/ERF [13], bZIP [14], DREB/ERF, MYB, NAC, WRKY [15], and GRAS [16].

GRAS TFs were first observed in bacteria and were classified in the Rossman folded methyltransferase superfamily taxon [17]. It was identified based on nuclear localization, DNA binding, and transcriptional activation characteristics [18]. It is named after three early identified functional genes, such as gibberellic acid insensitive (GAI), the repressor of GA1 (RGA), and scarecrow (SCR) [19]. The structure consists of 360-850 amino acids and contains a highly variable N-terminal and a highly conserved C-terminal structural domain [20]. The C-terminus contains five motifs, including LHRI, VHIID, LHRII, PFYRE, and SAW, constituting the GRAS domain [19]. The N-terminus contains two conserved DELLA and TVHYNP protein structures associated with GA signaling [21]. However, based on the conserved motifs and sequence similarity of the GRAS TF family, it was divided into ten subfamilies: DELLA, LS, SCR, SHR, PAT1, HAM, SCL3, SCL4/7, SCL9 (LISCL), and DLT [22]. It was found that the LISCL was named mainly from genes regulated by meiosis-related genes [23]. The DELLA subfamily contains *GAI*, *RGA*, and *RGL* genes involved in GA signaling processes, thereby indirectly regulating seed germination and flower development [24]. In contrast, SCL3 promotes GA signaling by antagonizing DELLA [25]. Interestingly, PAT1 acts primarily as a positive regulator of photosensitive pigment signaling and has a conserved motif in almost all members of the PAT1 subfamily that plays a vital role in light signaling [26].

Currently, with the development of genetics and genomics, genome-wide identification and analysis of the GRAS TF family has been completed in a variety of plants, including *A. thaliana* [27], *O. sativa* [28], *Populus* [29], pine [30], *Avena sativa* [31], and soybean [32]. The GRAS TF family has vital regulatory roles in plant growth and development [33]. Schumacher K et al. studied meristematic tissue development in tomatoes using a loss-of-function mutant of the LS gene and found that the gene in tomatoes can participate in the development of leaf axillary meristematic tissue and the formation of lateral buds [34]. The *SHR* gene can directly induce the SCR promoter in specific tissues, regulating the expression of *SCR* and *SCL23* and altering the developmental orientation of sheath cells [33]. In addition, the GRAS TF family is also involved in the plant response to adversity stress. Researchers found that the *PeSCL7* gene was overexpressed in *Populus* during the early stages of salt stress induction and that transgenic plants of this gene, *A. thaliana*, showed greater tolerance to salt and drought stress [35]. In *Halostachys caspica*, the expression of the *HcSCL13* gene gradually increased and differed significantly with increasing salt stress time compared to the control, indicating that this gene can respond positively to salt stress [36]. However, if plants are exposed to high salinity, ABA signaling is activated, which promotes the accumulation of DELLA proteins, inhibits plant growth, and enhances plant resistance [37]. Five and seven *MaGRAS* genes were upregulated in the root and aboveground parts of *Melilotus albus* under ABA, drought, and salt stress treatments, respectively [38]. In *Medicago sativa*, drought stress and hormone treatments (ABA, GA, and IAA) induced MsGRAS51 expression. However, expression was downregulated in salt-stress environments [39], suggesting that it plays a vital role in the response to ABA and abiotic stresses. However, although the GRAS family of TFs has been intensively studied as the so-called 'green revolution' genes for more than three decades now [40], an increasing number of GRAS family genes have been identified in plants, and their biological functions are gradually elucidated as the GRAS family genes have become better understood. However, there are no studies on the GRAS TF family in kiwifruit.

The purpose of our research was to comprehensively analyze members of the GRAS family in the whole kiwifruit genome for identification, physicochemical property analysis, phylogenetic analysis, gene structure, structural domains and conserved motifs, cis-acting regulatory elements, chromosomal localization, gene duplication, covariance analysis, GO and KEGG enrichment analysis, and protein network interaction analysis, as well as to explore kiwifruit expression patterns in response to salt stress. This study provides a basis for understanding the evolution and biological functions of the kiwifruit GRAS TF family and a fundamental basis for further research into breeding for resistance in kiwifruit under salt stress.

## Results

### Identification and physicochemical characterization of the kiwifruit GRAS TF family

In this study, 86 *AcGRAS* genes were finally identified by BLAST comparison and hmmsearch search (Additional file 1). They were also named *AcGRAS1-AcGRAS86* from top to bottom according to their position on the chromosome. Further analysis of the sequences showed that the protein lengths of the GRAS TF did not vary greatly, ranging from 409 (*AcGRAS86*) to 771 (*AcGRAS31*) amino acids, with molecular weights between 46.07 (*AcGRAS86*) and 86.24 (*AcGRAS46*) kDa, pI ranging from 4.74 *(AcGRAS63*)-7.23 (*AcGRAS42*), with instability coefficients ranging from 32.57 (*AcGRAS13*)-61.97 (*AcGRAS38*), particularly less than 40 for *AcGRAS13* only, indicating that all *AcGRAS* are unstable proteins except this protein. In amino acid composition, the average aliphatic index was 84, favoring the increased thermal stability of globular proteins. Subcellular localization predictions by the Plant-mPLoc tool showed that they were all in the nucleus (Additional file 2).

### Phylogenetic analysis of the GRAS TF family in kiwifruit

**Fig. 1:**
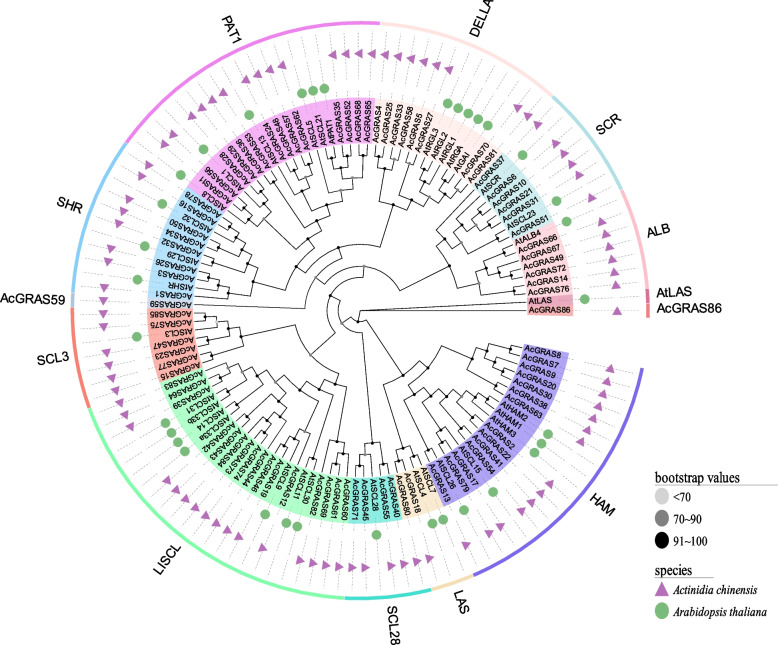
Phylogenetic tree of the GRAS TF family in *Arabidopsis thaliana* and kiwifruit. The phylogenetic tree was constructed using the maximum likelihood method and 1000 replicate bootstrap values. Black indicates 1000 replicates in which bootstrap values > 90, gray indicates 1000 replicates in which 90 > bootstrap values > 70, and white‒gray indicates bootstrap values < 70. This plant is labeled separately using different shapes and colors, with triangular purple for kiwifruit and round green for *A. thaliana*. Different groupings are indicated by different colors

To understand the evolutionary relationship of the kiwifruit *GRAS* TF family with another family. We used the ML method of MERGA and IQtree to construct phylogenetic trees for 86 GRAS protein sequences of kiwifruit and 34 of *A. thaliana*, and the results showed that the tree topologies were consistent (Fig. 1). Based on the clustering results, the kiwifruit GRAS TF family can be divided into ten subfamilies: HAM, LAS, SCL3, SCL28, LISCL, SHR, PAT1, DELLA, SCR, and ALB. AcGRAS59, AcGRAS86, and AtLAS each constitute a subgroup. The AcGRAS TF had the highest number in the LISCL subfamily, containing 23 TF family members, including 16 in kiwifruit and 7 in *A. thaliana*. In addition, 14, 2, 6, 8, 8, 14, 8, 6, and 6 AcGRAS TF family members were identified in the HAM, LAS, SCL3, SCL28, SHR, PAT1, DELLA, SCR, and ALB subfamilies, respectively.

### Analysis of gene structure, structural domains, and conserved motifs of the kiwifruit GRAS TF family

**Fig. 2:**
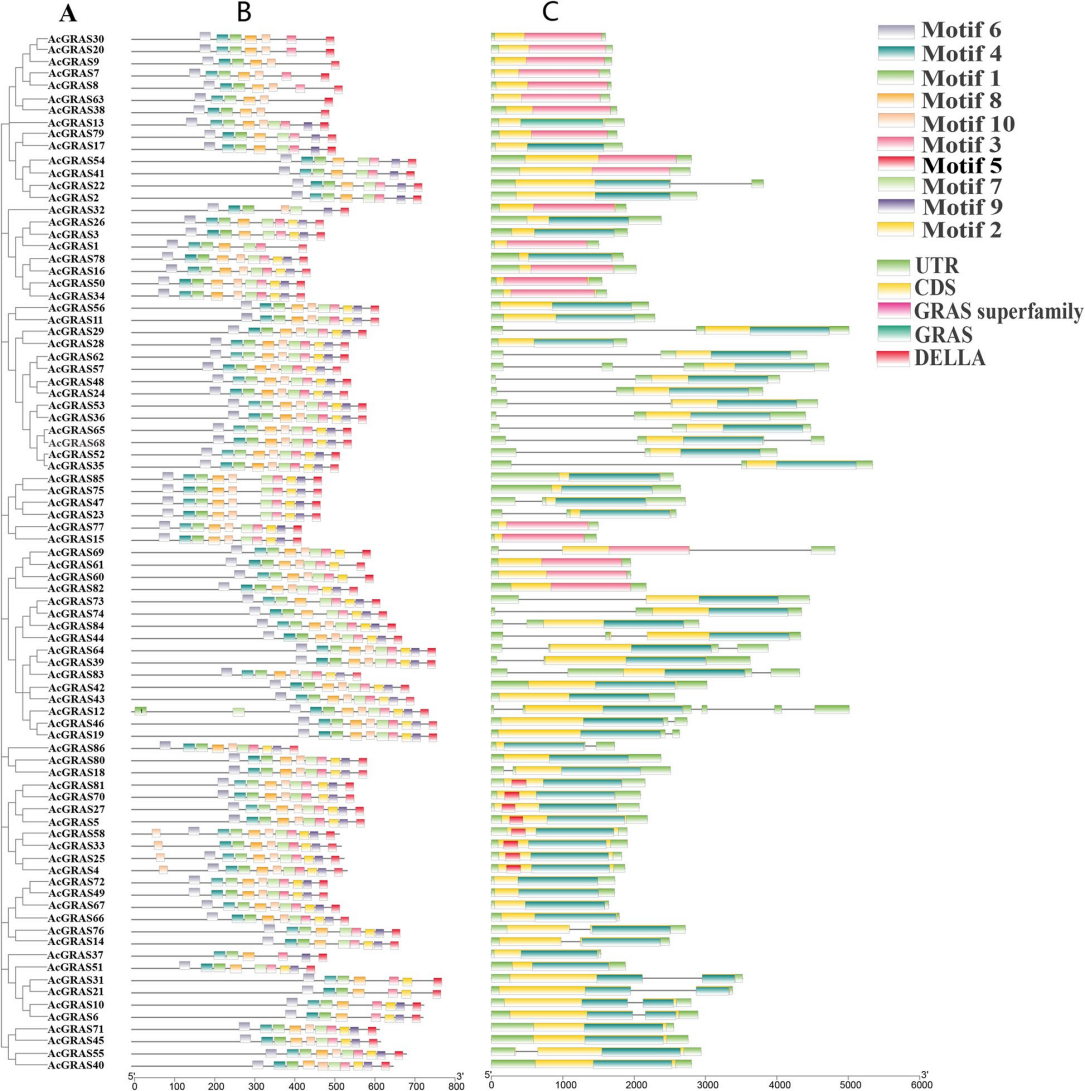
Phylogenetic relationships, gene structure, structural domains, and conserved motif distribution of *AcGRAS* TF family genes. **A** Phylogenetic analysis of 86 *GRAS* TF family gene constructs by MEGA. **B** Distribution of conserved motifs of AcGRAS, with motifs indicated by colored boxes and black lines indicating relative lengths of proteins. **C** Exon‒intron structures and conserved structural domains of *AcGRAS* genes

To further investigate the sequence characteristics of the AcGRAS protein, the motif composition of the protein was analyzed in this study using the MEME online tool (Fig. 2B). The results showed that conserved motifs 1, 2, 3, 4, 5, 6, 7, 8, and 9 were highly conserved and present in 95% of the protein sequences. However, the same motifs were in different positions in different protein sequences, which may be related to the structure and function of the protein. The results showed that conserved motifs 1, 2, 3, 4, 5, 6, 7, 8, and 9 were highly conserved and present in 95% of the protein sequences. However, the same motifs were in different positions in different protein sequences, which may be related to the structure and function of the protein. In contrast, motifs 1 and 7 appeared twice in the protein sequence of AcGRAS12 (Fig. 2A, B), suggesting that the *AcGRAS12* gene in the LISCL subfamily may be involved in a specific function. The distribution of motif 2 was not found in the HAM subfamily or AcGRAS37, which may be related to the unique evolutionary processes of this subfamily (Fig. 2A, B). The DELLA subfamily contains all motifs, and it can be assumed that the motif distribution of members of this subfamily is highly conserved. However, even members of the same subfamily have some differences in motif distribution, e.g., motif 10 appears twice in the protein sequences of AcGRAS58, AcGRAS33, AcGRAS25, and AcGRAS4 in the DELLA subfamily (Fig. 2A, B). This may be due to the differences between TF family genes under specific conditions.

Differences in the distribution of conserved motifs in the AcGRAS TF family may also be influenced by gene structure and structural domains. The distribution of gene structure is vital to evolutionary features within gene families, and the 86 *AcGRAS* TFs were analyzed for gene structure by sequence alignment and based on genome annotation files (Fig. 2A, C). The results suggest that the gene structure of most *AcGRAS* TF family genes is conserved. There were 0-4 introns in the 86 *AcGRAS* TF family members. Most subfamilies have no introns in the *AcGRAS* genes, except for the PAT1 and LISCL subfamilies, which contain 1-2 introns, and the *AcGRAS12* gene of the LISCL subfamily, which has four introns. The number of exons was relatively high in the PAT1 and LISCL subfamilies. However, all other subfamilies contained three exons, indicating that although there are some differences in the introns and exons of this TF family, their gene structure is also relatively conserved, especially in the subfamilies. In addition, the conserved structural domains were analyzed in this study, and the results showed that all 86 AcGRAS proteins have structural domains in the GRAS or GRAS superfamily and that the DELLA subfamily contains specific DELLA structural domains that play a vital role in signaling [41] (Fig. 2A, C). This further validates a correlation between conserved motifs, gene structure, and the distribution of structural domains with phylogenetic relationships, with more evolutionarily related members of the AcGRAS TF family having more similar sequence characteristics. Their structures were more identical than others.

### Analysis of cis-acting elements of the kiwifruit GRAS TF family genes

**Fig. 3:**
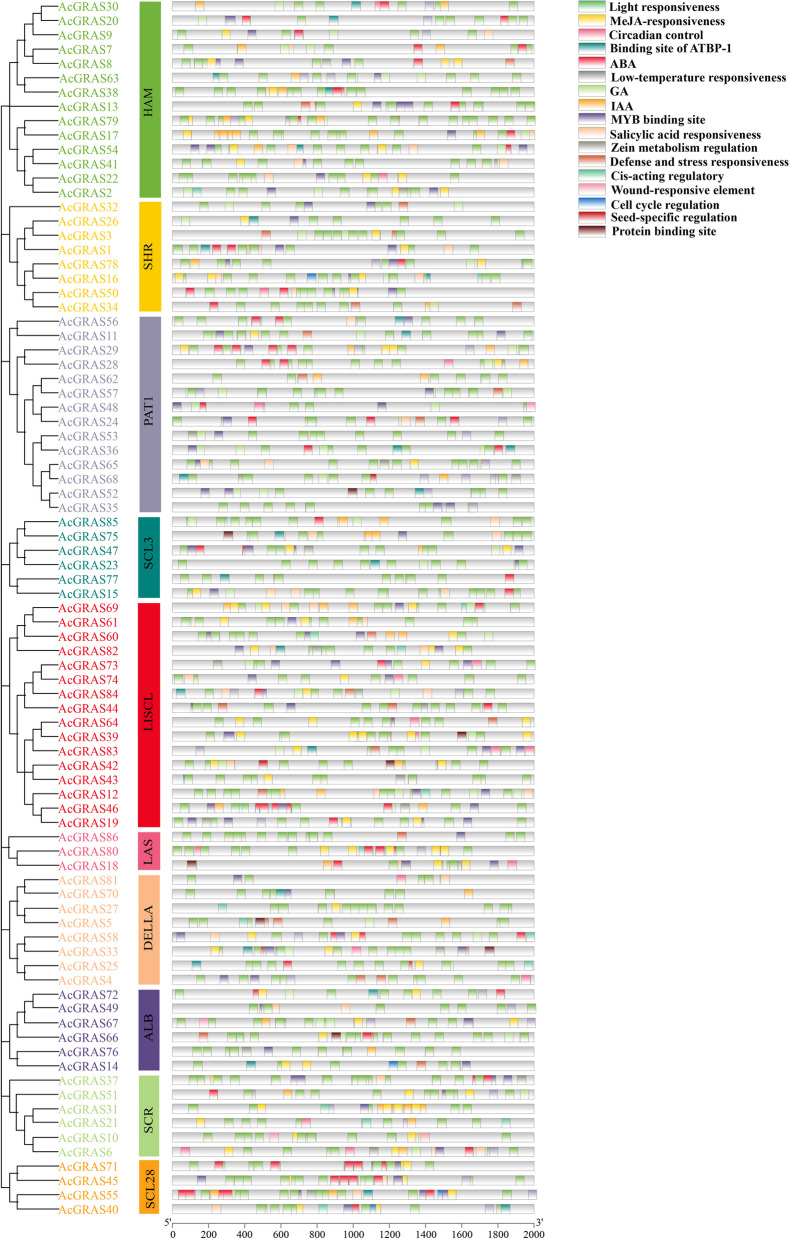
Distribution of cis-acting elements of the 2000 bp sequence upstream of the 86 *AcGRAS* TF family genes, with different cis-acting elements indicated by different colors

We analyzed the upstream 2000 bp sequences of 86 *AcGRAS* genes for cis-acting elements. The results demonstrate that the promoter region of each *AcGRAS* gene has multiple stress- or hormone-related cis-elements (Fig. 3). Of these, the light responsiveness element is present in almost every gene. The hormone-related cis-acting elements are second only to the light-responsive ones, e.g., MeJA abscisic acid (ABA), gibberellin (GA), indoleacetic acid (IAA), and salicylic acid. However, only five promoters of *AcGRAS* in the PAT1 subfamily appear in MeJA response elements, 64% of the promoters of genes in this subfamily have no distribution of MeJA response elements, and there are few, if any, IAA response elements in this subfamily. The presence of GA response elements was barely visible in the four subfamilies DELLA, SCL28, SCR, and ALB, except *AcGRAS5*, *AcGRAS58*, and *AcGRAS33* in the DELLA subfamily; *AcGRAS72*, *AcGRAS67*, and *AcGRAS66* in the ALB subfamily; and *AcGRAS51* and *AcGRAS6* in the SCR subfamily, where each gene contained only one GA response element except for the *AcGRAS6* gene, which had two GA response elements (Fig. 3). However, ABA-responsive genes are distributed relatively more in the SCL28 and PAT1 subfamilies than in the other subfamilies, which may result from the interaction of genes in this subfamily with ABA. It is followed by environmental stress response elements, e.g., low-temperature response and stress response elements. This type of response element is distributed in various subfamilies, such as *AcGRAS5* and *AcGRAS33* in the DELLA subfamily and *AcGRAS44*, *AcGRAS62*, *AcGRAS14*, and *AcGRAS60* in the LISCL subfamily. Other response elements were relatively less distributed, such as AT-enriched DNA-binding protein (ATBP-1) response elements, MYB binding sites, seed-specific expression regulatory elements, circadian rhythm response elements, etc. In particular, MYB binding sites were also equally distributed in the promoters of all *AcGRAS* TF family genes. In short, most promoter-cis-acting elements, except for the light response element, are involved in abiotic stresses and hormonal responses that regulate gene expression and substance metabolism in plants to make them resistant to stress, thereby improving the kiwifruit's ability to adapt under adverse conditions.

### Chromosomal localization, gene duplication, and covariance analysis of the GRAS TF family in kiwifruit

**Fig. 4:**
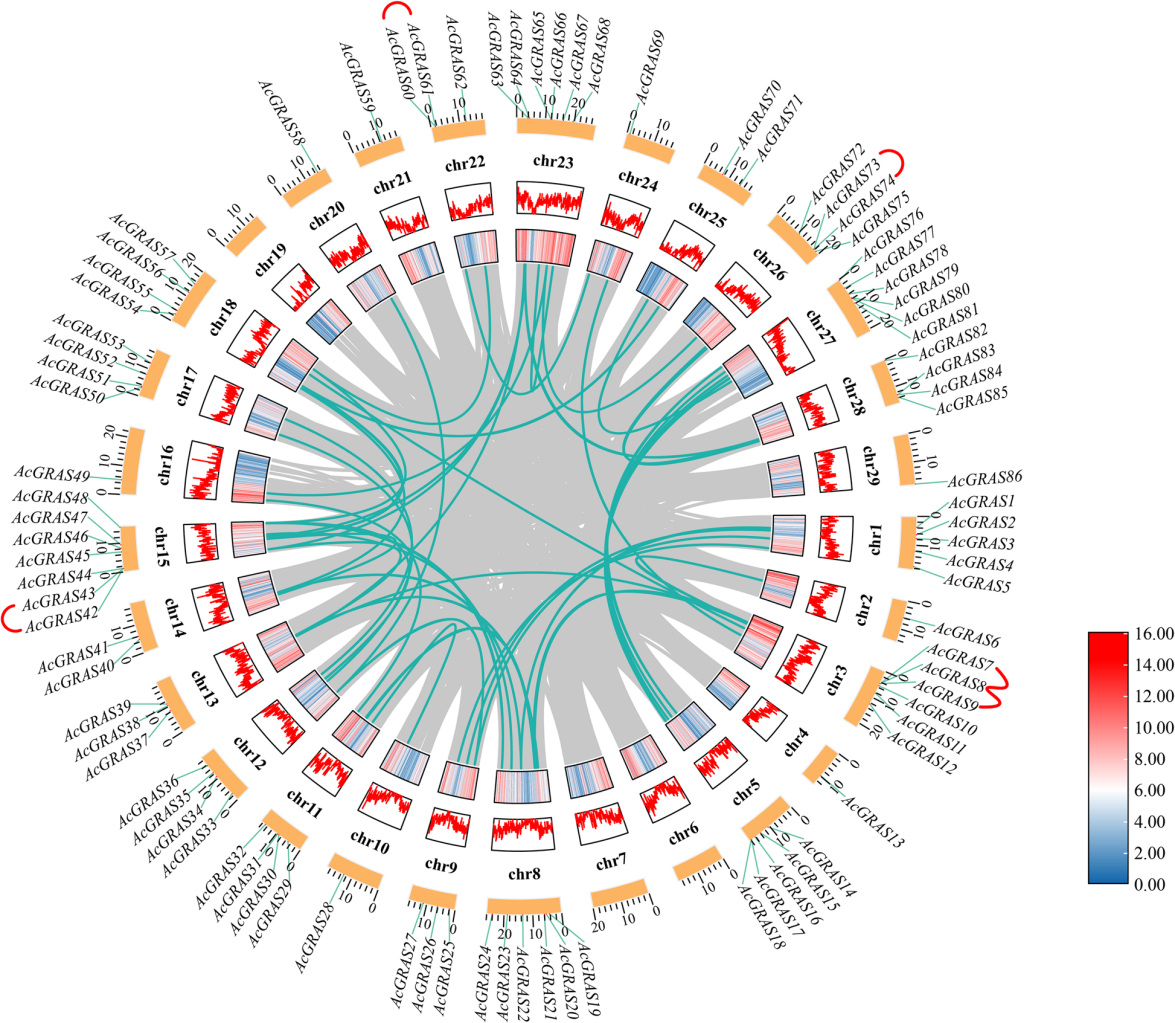
Covariance analysis of kiwifruit *AcGRAS* genes. Gray lines indicate all duplicated genes, dark green indicates fragmented duplicated *GRAS* gene pairs, and red lines connecting genes outside the chromosome indicate tandem duplicated pairs. The heatmap and line graph are gene densities, with line graph densities increasing from blue to white to red, and yellow rectangles are chromosomes, with chromosome names shown between each chromosome and gene density

This study used TBtools software to locate 86 *AcGRAS* genes on 29 chromosomes of the kiwifruit genome (Fig. 4) to understand the distribution and genome-wide density of kiwifruit *AcGRAS* genes on chromosomes. Our results reveal that there are no *AcGRAS* genes on chromosomes chr6, 7, and 19, only one *AcGRAS* gene on chromosomes chr2, 4, 10, 16, 20, 21, 24, and 29, and the highest number of *AcGRAS* genes distributed on chromosome chr15 with seven. The other chromosomes had 2-6 *AcGRAS* genes, which were unevenly distributed. There was also no direct relationship between chromosome length and the number of genes, e.g., chromosome 15 was 14 Mb long and had 7 *AcGRAS* genes, whereas chr16 was 20 Mb long and had only 1 *AcGRAS* gene.

Gene duplication events are essential for the evolution of family members. Fragment duplication and tandem duplication are the main drivers of gene duplication [42]. Eighty-six *AcGRAS* genes were analyzed for gene duplication (Fig. 4) to understand the replication events of the *AcGRAS* family genes in kiwifruit. The results showed that five tandem repeat gene pairs and 46 fragment repeat gene pairs were found, with tandem repeat genes distributed on chromosomes 3, 15, 22, and 26, all occurring close to each other. In contrast, fragmental duplicated genes were distributed on 18 chromosomes, with more genes distributed on chromosomes 5 and 8, while chromosomes 1, 3, 12, and 15 all contained four genes. These results suggest that fragment duplication may play a key role in gene duplication events in the AcGRAS family.

In addition, the GRAS covariance between kiwifruit, *A. thaliana,* and *O. sativa* was analyzed in this study (Additional Fig. 1). Our results suggest that kiwifruit and rice have 30 homologous pairs of genes, mainly on chromosomes 8 and 11. In contrast, there were 64 pairs of homologous genes with *A. thaliana*, with relatively more homologous genes occurring with higher frequency on chromosomes 5, 8, 15, 18, 25, and 27. This suggests that dicotyledons are evolutionarily more closely related to kiwifruit than monocots.

### Gene Ontology and Kyoto Encyclopedia of Genes and Genomes enrichment analysis of the *GRAS* TF family in kiwifruit

**Fig. 5:**
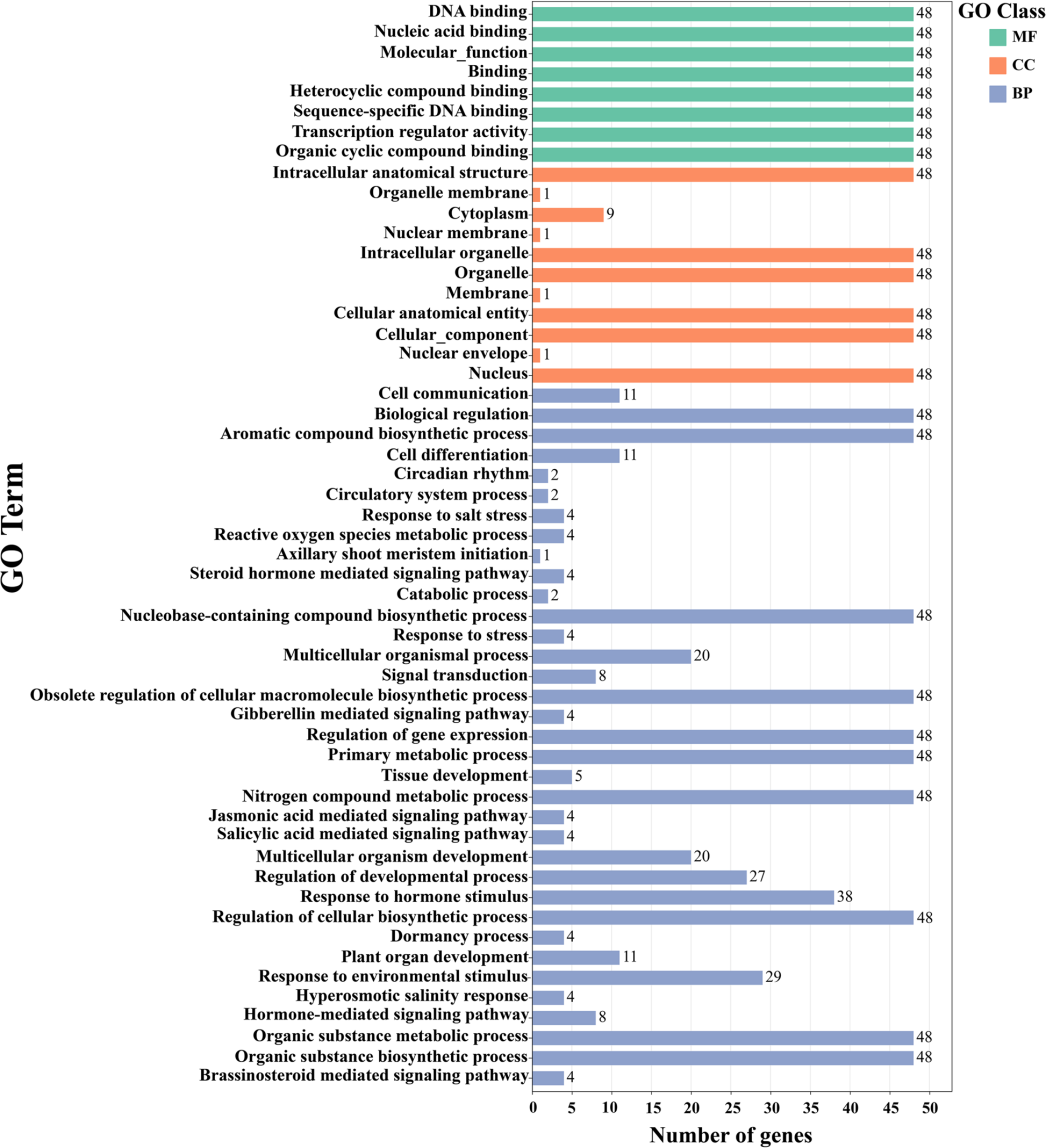
Results of the annotation of GO-Level 3 of the *AcGRAS* gene. MF denotes molecular function, CC denotes cellular component, and BP denotes a biological process, indicated in green, yellow, and blue, respectively. The x-axis presents the number of gene functions, and the y-axis is the annotated function of a gene

To further elucidate the biological functions of the *AcGRAS* TF family of genes, gene function annotation of the proteins of 86 *AcGRAS* genes was performed in this study. (Fig. 5, Additional file 3). The results showed that 48 *AcGRAS* genes out of 86 genes were annotated and assigned to three broad categories: molecular function (MF), biological process (BP), and cellular component (CC). In particular, the enrichment of *AcGRAS* genes for binding, nucleic acid binding, organic cyclic compound binding, and so on was higher among the molecular functions, indicating that the *AcGRAS* TF family genes play a vital role in molecular effections when performing regulation. However, among the cellular components, cells, intrinsic ingredients of cells, nuclei, and intracellular anatomical structures enriched for *AcGRAS* genes were higher in number. In biological processes, organic matter synthesis and metabolic processes, growth and development processes, responses to environmental stimuli, and responses to hormonal stimuli or mediated signaling pathways are enriched with more *AcGRAS* genes, among which hormonal stimulus responses, such as GA, salicylic acid and jasmonic acid-mediated (JA-Me) signaling pathways; environmental stimulus responses, such as responses to salt stress, hypertonic salinity responses, and other stress responses; and growth and developmental processes, such as plant organ development, dormancy processes, and tissue development, suggest that the *AcGRAS* TF family of genes may be adapting plants to different environments by regulating various parts of the plant. The number of genes significantly enriched in each prime category was greater than 48, suggesting that the same gene may play different roles in different functional entries.

In addition, KEGG enrichment analysis revealed (Additional file 4; Additional Fig. 2) that only 12 of the 86 *GRAS* genes were enriched for the sole plant hormone signaling pathway, namely, the *AcGRAS82*, *AcGRAS81*, *AcGRAS70*, *AcGRAS69*, *AcGRAS60*, *AcGRAS61*, *AcGRAS58*, *AcGRAS33*, *AcGRAS25*, *AcGRAS27*, *AcGRAS4* and *AcGRAS5* genes. *AcGRAS82*, *AcGRAS61*, *AcGRAS69*, and *AcGRAS62* are in the LISCL subfamily and belong to the GRAS superfamily. However, all other genes belong to the DELLA subfamily, and all are in this subfamily (Fig. 2). This finding may be related to the specific structure and function of the AcGRAS family of TFs.

### PPI network analysis of the GRAS TF family in kiwifruit

**Fig. 6:**
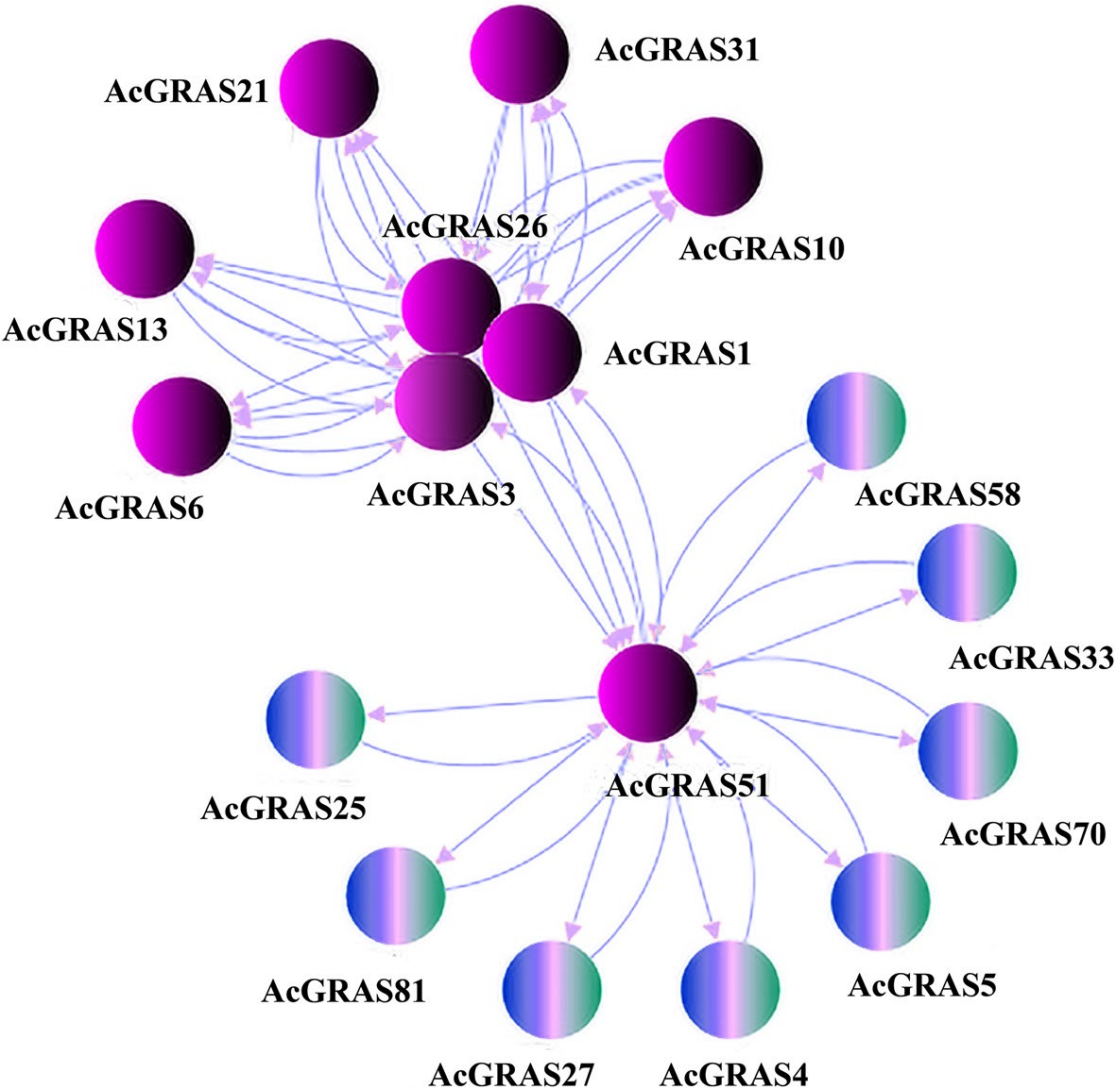
PPI reciprocal relationship diagram. Each circle represents a protein. The blue and green mixed-colored balls indicate genes enriched in the KEGG pathway for the hormone signaling process

Network interaction relationship analysis of 86 AcGRAS proteins (Fig. 6) showed that 17 genes formed interaction proteins, but 69 genes could not form interaction relationships. This indicates that these proteins have important roles, and eight colored proteins belong to the DELLA subfamily, a family of proteins known to interact with GA to regulate the adverse environment [24]. The other proteins belonging to the SCR and SHR subfamilies have relational interactions with them, especially the AcGRAS51 protein, which is central to linking DELLA proteins, indicating that its role is inseparable from the DELLA subfamily.

### Analysis of the expression pattern of *AcGRAS* TF family genes under salt stress

**Fig. 7:**
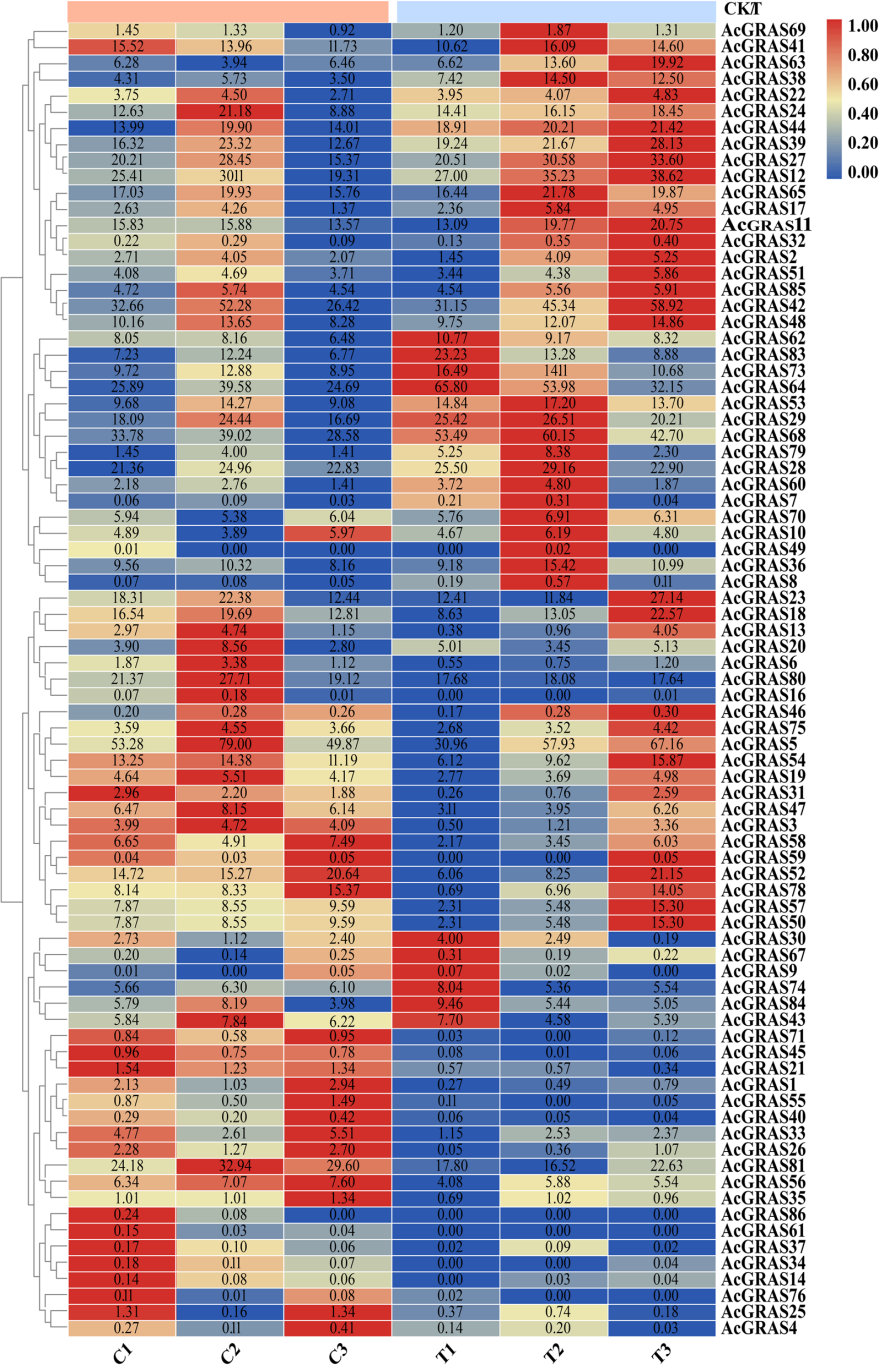
Heatmap of the expression profiles of 81 *AcGRAS* genes under salt stress. The different colored boxes indicate different log2 (FPKM) values, with expression gradually increasing from blue to yellow to red at a time. CK is the control group, and T is the treatment group

**Fig. 8:**
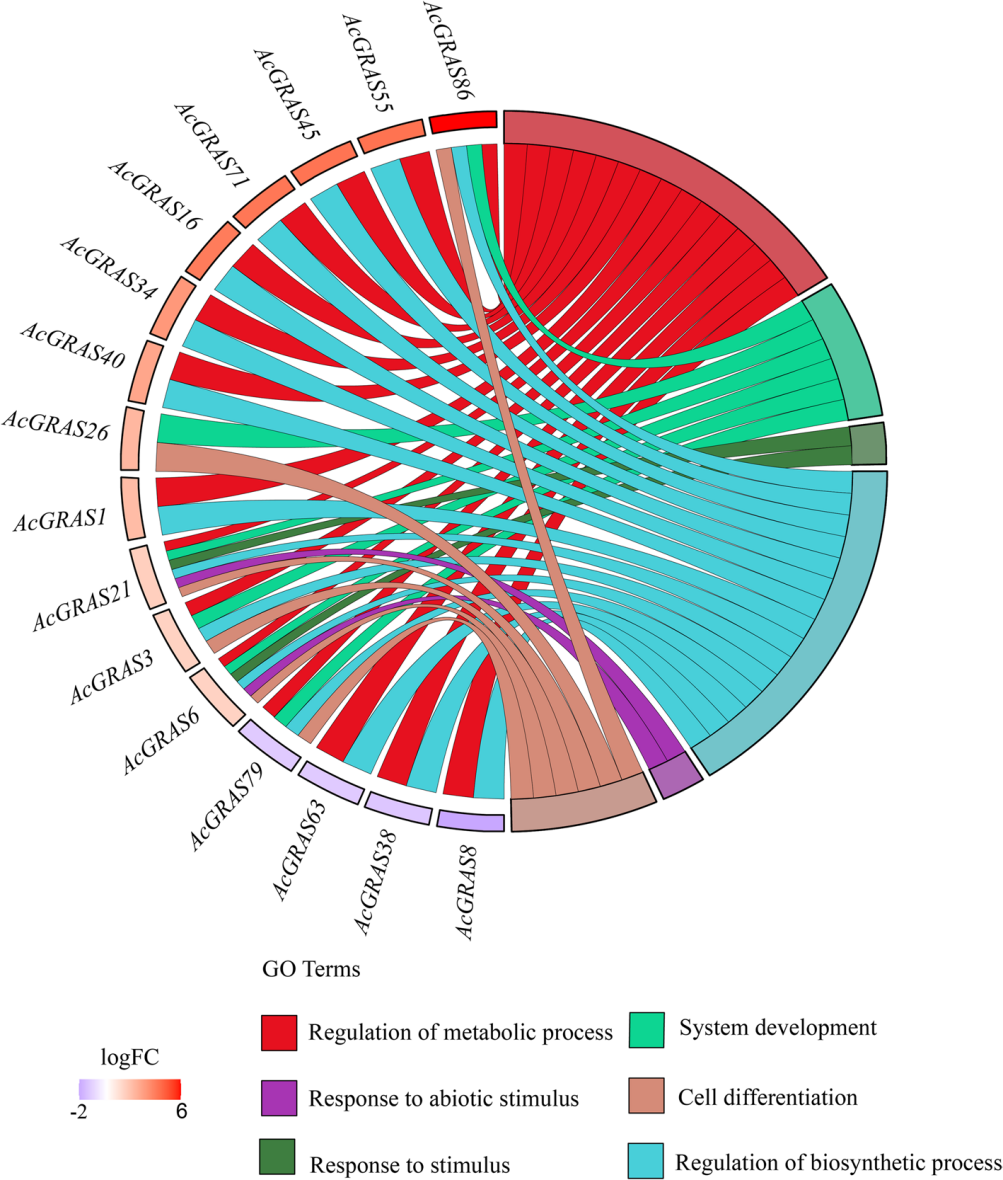
Expression pattern and functional annotation of 16 *AcGRAS* significantly differentially expressed genes under salt stress. The GO string diagram is divided into two parts, with genes on the left and arranged according to logFC and GO terms on the right, with different colors indicating different functions

**Fig. 9:**
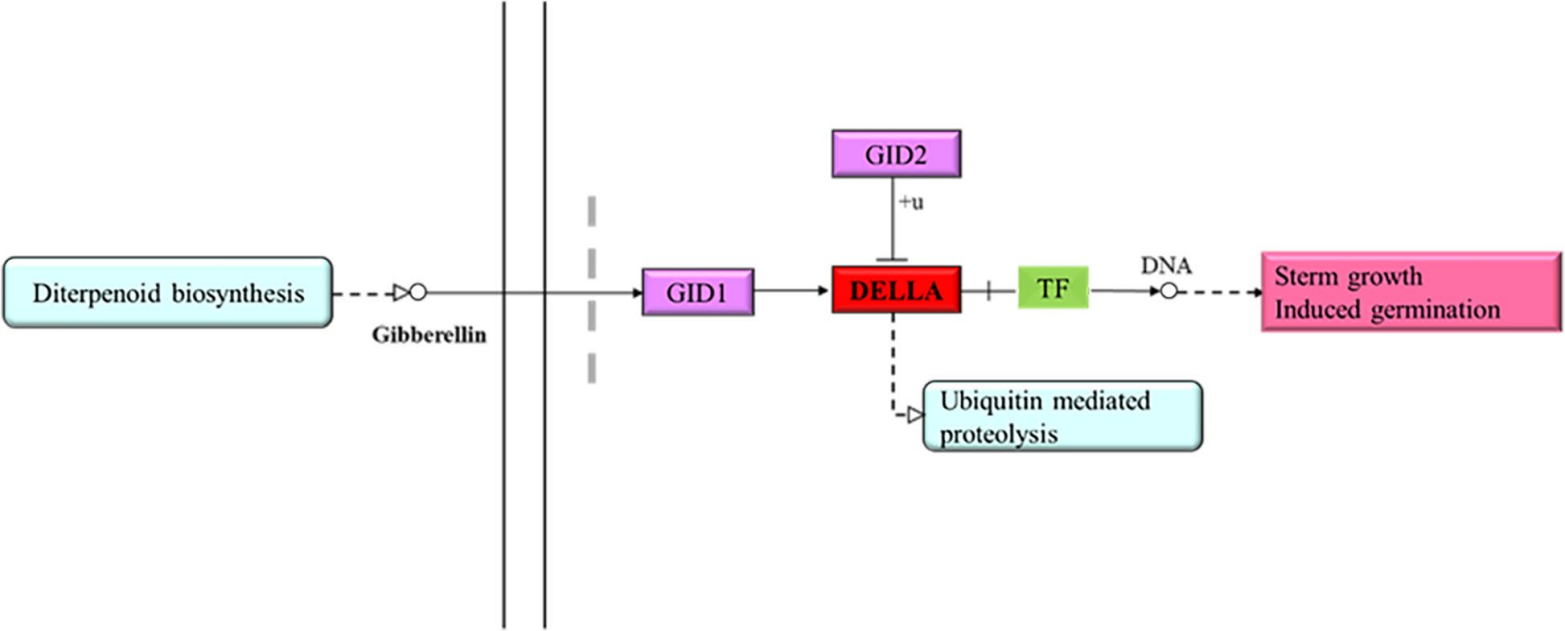
Diagram of the KEGG phytohormone signaling pathway of the *AcGRAS61* gene under salt stress. The figure is drawn with reference to the KEGG phytohormone signaling pathway map [43]. The red part of the diagram shows the regulation of the GA response by the DELLA protein

To understand the expression pattern of AcGRAS TFs in *A. chinensis* var. *deliciosa* cv. Xuxiang under salt stress, transcriptome data from *A. chinensis* var. *deliciosa* cv. Xuxiang treated with salt stress were analyzed in this study. The results showed that 81 *AcGRAS* expressed genes were identified; under salt stress, 41 showed downregulation, 40 showed upregulation, and 17 were differentially expressed significantly compared to the control (Fig. 7). To further understand the functions of the 17 genes with significant differences in *AcGRAS* expression in biological processes, GO string plots and KEGG enrichment analyses were conducted based on the results of GO and KEGG enrichment analyses described above as well as transcriptome data (Fig. 8; Additional files 3, 4; Additional file Fig. 2). The results showed that only the *AcGRAS8*, *AcGRAS38*, *AcGRAS63*, and *AcGRAS79* genes showed downregulation among the 17 differentially expressed genes, while the remaining 13 showed upregulation. The number of differentially expressed significant genes was significantly higher than the number of downregulated genes, indicating that the *AcGRAS* gene could improve the salt tolerance of the plants by positively regulating the damage caused to 'Xuxiang' kiwifruit during salt stress. However, this study also found that of the 17 genes significantly differentially expressed, all except *AcGRAS26* were involved in regulating metabolic and biosynthetic processes, suggesting that under salt stress, AcGRAS TFs in kiwifruit plants improve salt tolerance in plants mainly by adjusting their own metabolic and biosynthetic processes. Six *AcGRAS* genes play a role in plant phylogeny and cell differentiation, and *AcGRAS21* and *AcGRAS6* are associated with adversity stress and abiotic stress, possibly through gene upregulation to regulate the damage caused by salt stress to the plant. However, it is worth noting that the *AcGRAS61* gene has no GO annotation function but has a KEGG enrichment function. KEGG enrichment analysis (Fig. 9) showed that the *AcGRAS61* gene can encode the DELLA protein, a negative regulator of the GA response. Under salt stress, GA binding to GID1 facilitates the binding of GID1 to the N-terminal end of the DELLA protein, leading to a conformational change in the DELLA protein. The interaction between DELLA and GID2 is inhibited, and the ubiquitination process of the DELLA protein is prevented. DELLA and TF dissociation thus inhibit stem growth and development to regulate plant growth under adverse conditions.

### Expression analysis of AcGRAS under salt stress

**Fig. 10:**
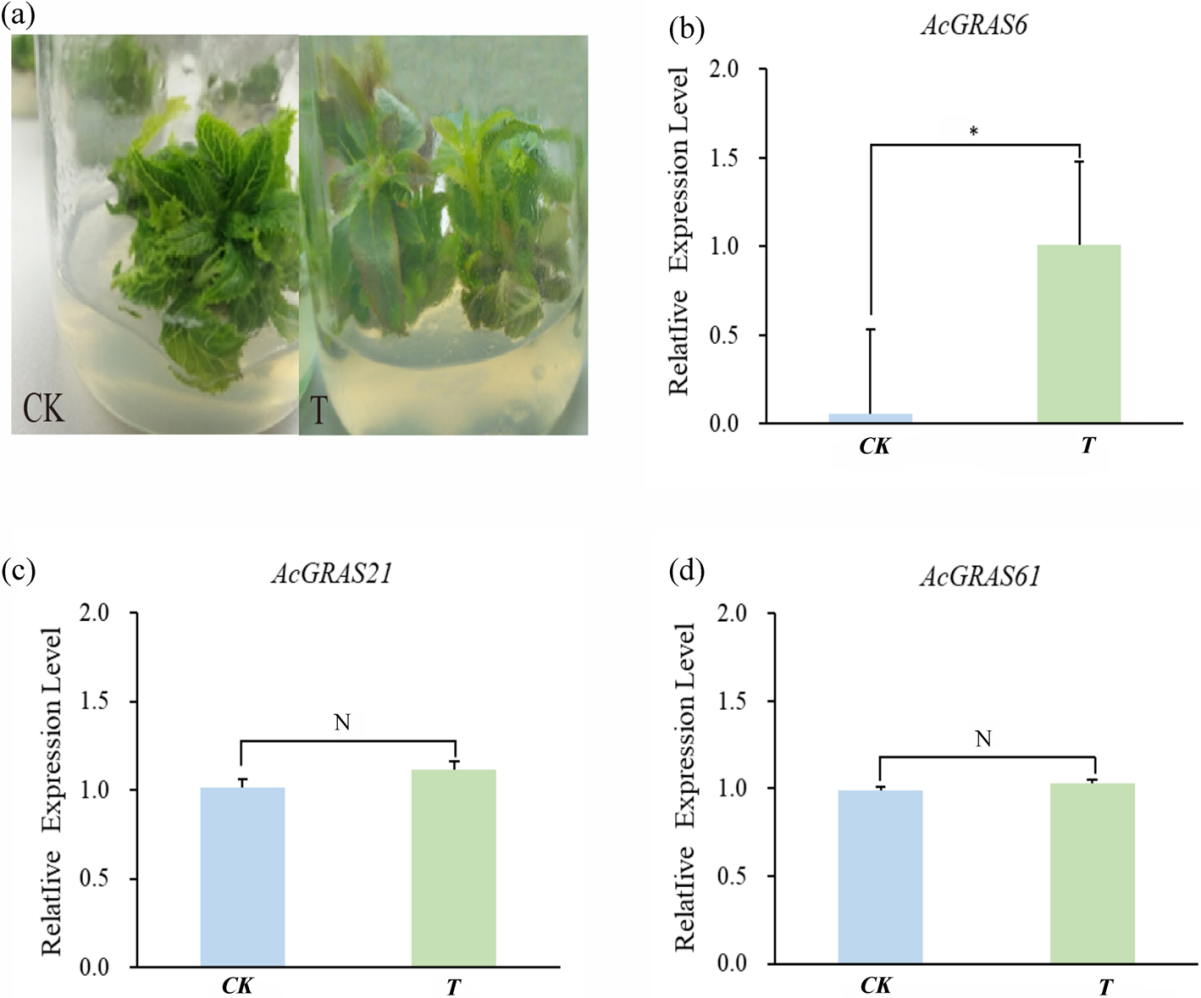
Expression analysis of *AcGRASs* under salt stress. **a** Response of kiwifruit to NaCl stress, **b** relative expression of *AcGRAS6*, **c** relative expression of *AcGRAS21*, and (**d**) relative expression level of *AcGRAS61* were determined by RT-qPCR. The standard error is expressed as an error bar. The data is expressed as the mean ± standard deviation of the three biological replicates. *indicates *p*-value <0.05; N indicate *p*-value >0.05; STUDENT's T test. CK is the control group, and T is the treatment group

To investigate the expression pattern of *AcGRAS* that may be associated with the salt response. The relative expression levels of the genes *AcGRAS6* and *AcGRAS21*, which are related to abiotic stress, and the *AcGRAS61* gene, which is related to the action of DELL proteins, were examined under salt stress using qRT-PCR. The results showed significant inter-phenotypic differences such as yellowing of leaves one week later of salt stress treatment, drying of leaves from the margins, and shrinkage of the margins of the more tender leaves (Fig. 10a). The qRT-PCR results showed that the significant differences in *AcGRAS6* expression under salt stress (Fig. 10b). Compared with the control, the expression of *AcGRAS21* (Fig. 10c) and *AcGRAS61* (Fig. 10d) was relatively increased, but the difference was not significant, which might be a result of the temporal and spatial differences of the treatments. It is noteworthy that after salt stress, the expression of *AcGRAS6* increased the most, and *AcGRAS21* and *AcGRAS61* already had slightly increased expression, suggesting that *AcGRAS61*, *AcGRAS6*, and *AcGRAS21* transcription factor genes have vital regulatory roles in plant response.

## Discussion

The GRAS family of TFs is widely present in plants and regulates a variety of adverse environments by mediating the expression of genes. Numerous studies have shown that the GRAS TF family plays a role in coping with abiotic stresses such as drought, salt, and low-temperature stress and regulates plant growth and developmental processes [16, 38, 44, 45]. 'Xuxiang' kiwifruit is one of the best varieties in terms of salt resistance [7]. The GRAS family of TFs has been intensively studied as the so-called "green revolution" genes for three decades [40]. An increasing number of *GRAS* family genes have been identified in plants, and the biological functions of *GRAS* TF family genes are gradually becoming understood. However, no genome-wide identification of the GRAS TF family in kiwifruit and analysis of their expression patterns in response to salt stress have been reported.

In the present study, a total of 86 *AcGRAS* genes were identified, which were relatively numerous compared to those of *A. thaliana* (34) and *O. sativa* (60) but lower than those of *Populus* (106) [29], soybean (117) [32] and wheat (153) [46], suggesting that the number of *GRAS* TF family genes correlates with genome size in different species. Phylogenetic analysis revealed that *AcGRAS59*, *AcGRAS86*, and *AtLAS* each form a separate branch, suggesting unique functions. The other *AcGRAS* genes clustered well with *A. thaliana,* and AcGRAS proteins were identified in each subgroup of *A. thaliana*, suggesting that these *AcGRAS* genes may have performed some essential biological functions during the long-term evolution of kiwifruit [29]. The GRAS TFs of kiwifruit can be divided into ten subfamilies, of which LISCL is the largest. However, the PAT1, HMA, DELLA, and SHR subfamilies contain several genes, but there is still some variation. The results are similar to the phylogenetic analyses of *M. albus* [38], sweet potato [47], *A. thaliana*, *O. sativa,* and *Populus* [29]. In addition, the classification was more evident in the combined conserved motif, gene structure, and structural domain analyses. In the conserved motif analysis, similar genes were found to cluster in the same subclade, and multiple motifs were present in specific proteins, implying that they may have unique functions [48]. We found that the DELLA domain is only in the DELLA subfamily, while the GRAS superfamily is only in the SHR, HAM, and LISCL subfamilies, which may be responsible for the diversity of the *GRAS* gene family and influence its functional differentiation. More interestingly, analysis of gene structure showed that most of the genes were intronless, except for the *AcGRAS* genes of the PAT1 and LISCL subfamilies, similar to the *GRAS* genes in sweet potato [47], *A. thaliana* [29], *Prunus mume* [49], grapevine [31] and potato [50]. However, intron-less genes tend to respond quickly to changes in environmental conditions [51], and therefore, many *AcGRAS* genes may also have similar functions. There are exceptions, such as the *AcGRAS12* gene of the LISCL subfamily, which contains four introns and differs markedly from other *AcGRAS* genes, possibly chromosomal structural variation, which may lead to functional diversity in the gene family. The clustering of phylogenetic trees with highly similar structural features of conserved motifs, gene structures, and structural domains further suggests that genes with similar evolutionary processes may also have similar functions, thus facilitating the screening of *GRAS* genes with similar functions. For example, heterologous overexpression of *HcSCL13* in *Halostachys caspica* enhanced plant growth and salt tolerance in transgenic *A. thaliana* [45]. The *A. thaliana SCL15* gene increased drought tolerance [52]. It can be hypothesized that PAT1 and *AcGRAS* genes of the HAM subfamily have similar functions. The *GRAS* gene LeHAM3 in the HAM subfamily of tomato studies also showed a higher response to salt stress at the seedling stage [53]. Additionally, transgenic *Populus and A. thaliana *plants of the *PeSCL7* gene showed greater tolerance to salt and drought stress [35]. SCL14 in the LISCL subfamily was more responsive to salt stress in *A. thaliana* [54]. It can be hypothesized that *AcGRAS* genes play a vital role in regulating abiotic stresses, especially salt stress [55].

Cis-acting elements play a vital role in the transcription of neighboring genes. In this study, the main cis-acting elements in the initiation region of the AcGRAS TF family were light-responsive cis-acting, hormone-related cis-acting, and environmental stress-responsive elements. There are numerous cis-acting hormones, such as GA, ABA, and salicylic acid. Eighty percent of the gene promoters contain GA response elements, except for a few genes. ABA response elements are relatively well distributed in the SCL28 and PAT1 subfamilies. Studies have shown that the N-terminal DELLA protein structure of the GRAS transcription factor family is involved in GA signaling and thus regulates plant growth and developmental processes [56]. GA levels or signaling can also affect salt tolerance in plants under salt stress [57]. DELLA inhibitory factor deletion reduces salt tolerance in *A. thaliana* [37]. Whereas ABA generally promotes stem elongation, flower development, and seed germination, plants deficient in ABA will have reduced cellular activity, and the factor activity related to ABA synthesis is enhanced under adversity stress [58]. For example, high salt stress can activate ABA signaling, thereby promoting the accumulation of DELLA proteins, inhibiting plant growth, and enhancing resistance [37]. This suggests that GRAS TFs in kiwifruit also play a vital role in regulating plant growth and development under adverse stress conditions. GO enrichment analysis revealed that hormonal stimulus responses, such as ABA, salicylic acid, and jasmonic acid-mediated signaling pathways; environmental stimulus responses, such as responses to salt stress, hypertonic salinity responses and other stress responses; and growth and development processes, such as plant organ development, dormancy processes, and tissue development, were enriched with a high number of genes. In contrast, only one phytohormone signaling pathway was identified in the KEGG enrichment analysis, which further suggests that AcGRAS TFs regulate plant growth and development by adjusting the expression levels of hormones in response to the stressful environment. The results further supported the results of the PPI network analysis.

Gene duplication is the evolutionary force behind the formation and expansion of the *GRAS* gene family [59]. In the present study, all *AcGRAS* genes were distributed unevenly on 26 chromosomes; three chromosomes did not contain *AcGRAS* genes, and most genes were located on chromosome 15. Interestingly, tandem duplication genes were on chromosomes 3, 15, 22, 24, and 26. In addition to tandem duplication, most genes were derived from fragment duplication genes, suggesting that fragment duplication is a prime force in gene family expansion. This result is similar to the results of four beans [60], cucumber [61], and *Populus* [35]. In addition, this study further compared the covariance of kiwifruit with one dicotyledonous plant and one monocotyledonous plant. The covariance of kiwifruit with the dicotyledons was superior to that with the monocotyledons.

When plants are subjected to adversity stress, they respond by corresponding vital activities that maintain normal intracellular activities, where significantly different genes may be involved in the corresponding response pathways [62]. Studying the expression patterns of relevant genes based on transcriptome data is a novel approach. In the present study, the expression pattern of *AcGRAS* genes under salt stress treatment was investigated, and 17 differentially and significantly expressed genes were identified, with 13 *AcGRAS* genes upregulated and four downregulated compared to the control. The upregulated genes belonged to the SCL28, SHR, and SCR subfamilies, except for *AcGRAS86*, a phenomenon also present in the root systems of *Citrullus lanatus* [63] and soybean [32]. In contrast, the downregulated *AcGRAS8*, *AcGRAS79*, *AcGRAS63*, and *AcGRAS38* belong to the HAM subfamily, which regulates the growth and development of plant roots [64], and in plant stem development, HAM affects the lateral organ primordia and stem-vascular tissue development by encoding GRAS proteins [65]. This suggests that the SCL28, SHR, SCR, and HAM subfamily genes play vital regulatory roles under salt stress. To better understand the function of these DEGs, this study found that of the 17 genes significantly differentially expressed, all were involved in regulating metabolic and biosynthetic processes except *AcGRAS26*, suggesting that the *AcGRAS* TF in kiwifruit plants may improve salt tolerance in plants under salt stress by adjusting their own metabolic and biosynthetic processes. Notably, the *AcGRAS86* gene forms a separate branch in the phylogeny, and it has elevated expression under salt stress, possibly due to the functional specificity of this gene in kiwifruit, which plays a vital role in adversity stress regulation. This is also reflected in *O. sativa*, where the *OsGRAS23* gene acts as a specific gene to induce drought resistance in *O. sativa* [66]. The *MaGRAS33* gene can significantly increase the tolerance of the white aromatic herb Miscanthus after salt treatment [38]. This suggests that in addition to gene gene interactions that regulate plant growth in response to adversity, individual genes can also play specific regulatory roles in different plants, thereby improving plant stress tolerance. Analysis of the GO flashy map and KEGG enrichment results also indicated that the *AcGRAS61* gene is specific, encoding a negative regulator of the GA response, the DELLA protein. It maintains GA levels to regulate stem growth and development in response to salt stress. It has been verified in some plants, such as *A. thaliana* [37, 67] and cereal crops [68], and in some review articles [56, 69].

## Conclusion

Eighty-six AcGRAS TFs were identified from the kiwifruit genome, and phylogenetic analysis classified them into ten subclades. Eighty-one *AcGRAS* genes were found to be expressed in the transcriptome data under salt stress. Of these, 17 *AcGRAS* genes were significantly differentially expressed compared to the control group. Among these 17 genes, most of the upregulated *GRAS* genes, such as *AcGRAS86*, *AcGRAS55,* and *AcGRAS69,* were enriched in GO biosynthesis and regulation of substance metabolism, indicating that their upregulated *GRAS* genes can reduce salt damage to kiwifruit plants by positively regulating salt stress and plant metabolism and biosynthesis, thus improving the salt tolerance of the plants. However, it is noteworthy that among these differentially expressed genes, the *AcGRAS61* gene is specific, encoding a negative regulator of the GA response, the DELLA protein. It maintains GA levels to regulate stem growth and development in response to salt stress.

qRT‒PCR analysis showed that the expression of *AcGRAS21*, *AcGRAS6,* and *AcGRAS61* was elevated under salt stress. Our results may provide a reference for future development of abiotic stress-tolerant transgenic kiwifruit and provide a basis for future exploration of the characteristics and functions of more *AcGRAS* genes.

## Materials and methods

**Fig. 11:**
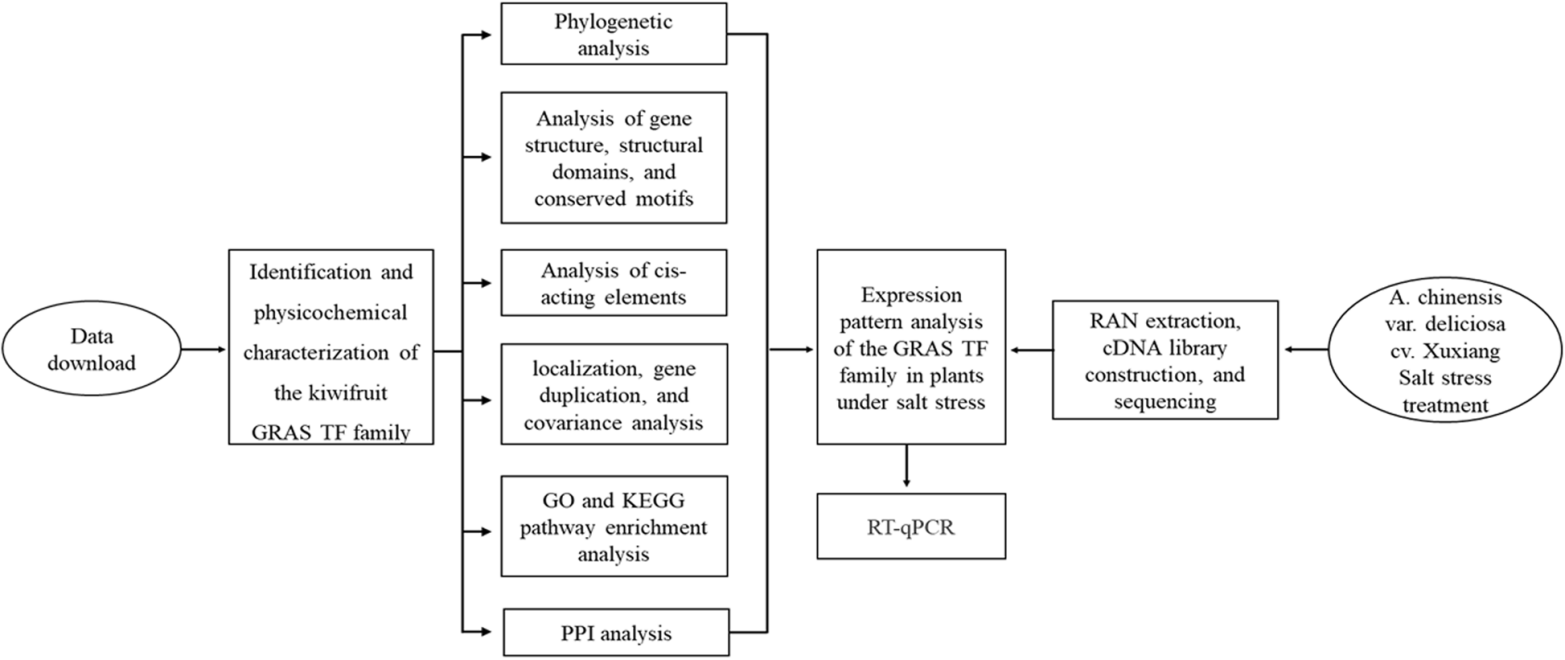
Flowchart of the experimental methodology of this study, where shapes indicate specific content steps and arrows indicate next steps

The kiwifruit variety used in this study was *A. chinensis* var. *deliciosa* cv. Xuxiang introduced from Northwest A&F University. For this variety, the treatment material was obtained by group culture using the method of Zhao et al. [70]. First, based on kiwifruit genomic data, the GRAS transcription factor family was analyzed using bioinformatics methods. Second, the material obtained above was used for salt stress treatment, and the treated kiwifruit was subjected to transcriptome analysis to obtain the significantly different *AcGRAS* genes. Finally, the above analysis results were combined to screen the genes with significant differences for qRT-PCR validation, and the main steps are shown in the flowchart (Figure 11).

### Data and Materials used in this study

The kiwifruit genome data (Red5_PS1_1.69.0) from the Kiwifruit Genome Database KGD: Kiwifruit Genome Database (http://kiwifruitgenome.org/) and sequence information for the conserved structural domain of the GRAS TF family [71, 72] (ID: PF03514) were downloaded from the Pfam website (http://pfam.xfam.org/). Protein sequences and genomic data for the 34 GRAS TF families in *A. thaliana* were downloaded from the *A. thaliana* genome website TAIR (https://www.Arabidopsis.org/) [27]. Genomic data for *O. sativa* were downloaded from the JGI (https://phytozome-next.jgi.doe.gov/info/OsativaKitaake_v3_1) website [73].

### Identification and physicochemical characterization of the kiwifruit GRAS TF family

Based on the results of the data downloaded above, the GRAS TF family conserved structural domain GRAS (ID: PF03514) in the kiwifruit genome protein database was searched using the hmmsearch program of HMMER 3.0 software, and protein sequence numbers with E-value <1 × 10-5 were filtered from the results [74]. Second, the protein sequences of the *A. thaliana* GRAS transcription factor family were blasted by the BLASTP program of Blast software, and the protein sequences of the kiwifruit genomic protein data with high homology (E-value <1 × 10-5) with the protein sequences of the *A. thaliana* GRAS TF family were screened [27, 75]. Then, the above steps were repeated to confirm the results. Finally, the sequence numbers from the above steps were merged. The merged protein sequence numbers were extracted from the kiwifruit genomic protein data using the TBtools tool to obtain the corresponding sequence and saved as files in the fast form [76]. Using the Conserved Domain Database (CDD) tool of the NCBI database (https://www.ncbi.nlm.nih.gov/Structure/cdd/wrpsb.cgi) and the online software PFAM (http://pfam.xfam.org/search#tabview=tab1), the presence of GRAS structural domains was further confirmed [77]. The GRAS TF family members of kiwifruit were obtained by removing the structural domain sequences that were not present or incomplete.

The identified GRAS TFs were also renamed according to their chromosomal location by the nomenclature using GRAS + sequence number. Physicochemical properties were analyzed using ProtParam (http://www.expasy.org/tools/protparam.html) online software for molecular weight (MW), theoretical pI, amino acid number, and instability coefficient (aliphatic index) [78]. Subcellular localization was performed using the Plant-mPLoc (Plant-mPLoc server (sjtu.edu.cn)) online tool.

### Phylogenetic analysis of the GRAS TF family in kiwifruit

The evolutionary relationships between the kiwifruit and *A. thaliana* GRAS TF families were analyzed. The GRAS protein sequences of kiwifruit and *A. thaliana* obtained from the identification were combined, sequence compared using MEGA11.0 software, trimmed using TBtools software, and then a phylogenetic tree was constructed using MEGA11.0 software and IQtree's maximum likelihood method (ML) [79]. The evolview (http://www.evolgenius.info/evolview/#/treeview) online software was also selected to visualize the phylogenetic tree. Finally, 86 kiwi GRAS proteins were grouped according to the grouping of *A. thaliana* GRAS proteins [80].

### Analysis of gene structure, structural domains, and conserved motifs

We chose TBtools software to analyze the gene structure of each gene based on the genome annotation file (GFF3) of kiwifruit [76]. Their structural domains were then predicted using the NCBI database's CD-search online tool (http://www.ncbi.nlm.nih.gov/Structure/bwrpsb/bwrpsb.cgi) with default parameters [81]. The conserved motifs of GRAS proteins were also analyzed using MEME (http://meme-suite.org/) online software [82], thus finding the differences between AcGRAS family members. Finally, the above results were visualized by TBtools software [76].

### Analysis of cis-acting elements

Based on the identification of the obtained gene IDs, we extracted the 2000 bp upstream base sequences of each gene separately from the genome annotation file (GFF3) using TBtools software, and we then used the online analysis software PlantCARE (https://bioinformatics.psb.ugent.be/webtools/plantcare/html/) to analyze them for promoter cis-acting elements [83].

### Chromosome localization, gene duplication, and covariance analysis

Chromosome position information was obtained from the genome annotation file (GFF3), and chromosome positions were mapped using TBtools software [76]. Gene duplication events were analyzed using MCScanX, and covariance between the GRAS transcription factor family members in *A. thaliana*, *O. sativa,* and kiwifruit was determined using TBtool software [84].

### Gene Ontology function of the *GRAS* gene and Kyoto Encyclopedia of Genes and Genomes pathway enrichment analysis

The protein sequences of the AcGRAS TF family were extracted from kiwifruit whole genome protein sequence files, and the proteins of 86 *AcGRAS* genes were annotated for gene function through the EggNOG-MAPPER database (http://eggnog-mapper.embl.de/) [85]. Gene Ontology function (GO) of the *GRAS* gene and Kyoto Encyclopedia of Genes and Genomes (KEGG) pathway enrichment analysis were then performed on the *GRAS* genes of kiwifruit using TBtools. It was also mapped and visualized by the online tool ChiPlot (https://www.chiplot.online/bar_plot_width_category) [78].

### Protein-protein interaction network analysis

The STRING protein‒protein interaction (PPI) database (http://string-db.org/), which records protein interactions for most species, was used. A network interaction map was constructed for 86 GRAS proteins in kiwifruit. They were then embellished using Cytoscape_v3.9.1 software, and the individual proteins that could not form interactions no longer appeared.

### Analysis of the expression pattern of AcGRAS in response to salt stress

#### Salt stress treatment

Fresh and healthy leaves of this variety were selected as explants to obtain healing tissues by histopathic techniques, and all media involved in the induction described below were adopted by Zhao et al. [70]. NaCl concentrations were set at 0 mmol/L, 34 mmol/L to 273 mmol/L for the nine treatments [86], and the 95% lethal concentration of NaCl for the healing tissues was 170 mmol/L in our experiments [87]. Three plants were then placed in salt proliferation mediums containing 170 mmol/L NaCl, denoted as T_1, T_2, and T_3, and three plants were placed in salt-free proliferation mediums, denoted as C_1, C_2, and C_3. One week later, leaves from the plants were taken and stored in liquid nitrogen at -80°C for RNA extraction and transcript sequencing.

#### RNA extraction, cDNA library construction, and sequencing

In this experiment, the total RNA of the samples was extracted using the kit method, and then eukaryotic mRNA was enriched with magnetic beads with oligo(dT). Next, mRNA was fragmented using a fragmentation buffer. cDNA first strand was synthesized using fragmented mRNA as a template with a six-base random primer, and the cDNA second strand was synthesized by adding buffer, dNTPs (A, U, G, C), RNase H, and DNA polymerase I. After purification by the QiaQuick PCR kit and elution with EB buffer, end repair, the addition of poly(A), and ligation of sequencing junctions, fragment size selection by agarose gel electrophoresis and PCR amplification were carried out to complete the whole library preparation. The constructed libraries were sequenced using the Illumina NovaSeq 6000 platform with the PE150 sequencing strategy. For sequencing, the material was removed from liquid nitrogen, placed in dry ice, and handed over to Microsegmentation Genetics for transcriptome sequencing. The results were uploaded to NGDC (Accession number CRA002280, https://ngdc.cncb.ac.cn/search/?dbId=gsa&q=CRA002280).

#### Expression pattern analysis of the GRAS TF family in *A. chinensis* var. *deliciosa* cv. Xuxiang plants under salt stress

The whole genome of Chinese kiwifruit (http://kiwifruitgenome.org/organism/3) was selected as the reference genome for the transcriptome sequencing data, and Bowtie2 software was used for sequence alignment. Then, using RSEM v1.3.0 [88] software, Bowtie2 was called to tally the comparison results to obtain the number of Reads for each sample compared to each transcript, and the average expression level (Fragments Per Kilobase per Million bases FPKM) conversion was calculated and performed for each transcribed region. The paired-end reads from the same fragment were counted as one fragment, which resulted in expected count data for gene and transcript expression levels, where genes with FPKM values greater than 0 were considered to be expressed genes. Differential gene detection was performed on the expected count data using a Poisson distribution method (Passion Dis). The *p*-value of the assay was corrected for multiple hypothesis testing [89]. The domain value of the *p*-value was determined by controlling the FDR (false discovery rate) [90]. The read count information was then used to perform differentially expressed gene analysis using the R language package edge R. Genes with a threshold of FDR < 0.05, log2FC > 1, or log2FC < -1 were screened as the final DEGs (Differentially expressed genes) [75].

#### Real-time fluorescence quantitative PCR

To further validate the expression of the specific *AcGRAS61* gene and *AcGRAS21* and *AcGRAS6* related to adversity stress and abiotic stress under salt stress. Total RNA was extracted from all samples using the RNA Extraction Kit and reverse transcribed into cDNA for qRT-PCR analysis using the HiScript 1st Strand cDNA Synthesis Kit. Primer 6.0 was then used to design qRT-PCR primers (Additional file 5). The *Actin* gene was used as an internal reference gene, and standard qRT-PCR was performed using a SYBR Green qPCR Master Mix (Universal) kit with at least three replicates for each gene [91]. Finally, the 2 ^-ΔΔCt^ method was used for analysis.

### Statistical analysis

Statistical differences between treatments were determined by t-tests using SPSS version 21 (*p*-values less than 0.05 are indicated by *; *p* values more than 0.05 are indicated by N). Data are presented as the mean ± standard deviation of three independent biological replicates. Graphs were produced using Excel.

### Supplementary Information

**Additional file 1:** **Table 1.** Results of AcGRAS TF family identification.

**Additional file 2:** **Table 2.** Analysis of physicochemical properties of AcGRAS TFs.

**Additional file 3:** **Table 3.** The GO enrichment analysis of the GRAS genes.

**Additional file 4:** **Table 4.** The KEGG Pathway enrichment analysis of the GRAS genes.

**Additional file 5.** The primers information for quantitative real-time PCR.

**Additional file 6:** **Additional Fig 1.** Covariate distributions in kiwifruit, Arabidopsis thaliana and rice.

**Additional file 7:** **Additional Fig 2.** AcGRAS gene KEGG is enriched to the KEGG pathway.

## Data Availability

The kiwifruit genome data (Red5_PS1_1.69.0) were downloaded from the Kiwifruit Genome Database KGD: Kiwifruit Genome Database (http://kiwifruitgenome.org/). RNA-Seq data were presented at the Genome Sequence Archive of the Beijing Institute of Genomics (BIG) Data Center (accession number CRA002280, https://ngdc.cncb.ac.cn/search/?dbId=gsa&q=CRA002280). Protein sequences and genomic data for the 34 GRAS TF families in *A. thaliana* were downloaded from the *A. thaliana* genome website TAIR (https://www.Arabidopsis.org/). Genomic data for *O. sativa* were downloaded from the JGI (https://phytozome-next.jgi.doe.gov/info/OsativaKitaake_v3_1)) website.

## Abbreviations

TF: Transcription factor
GO: Gene Ontology
KEGG: Kyoto Encyclopedia of Genes and Genomes
MW: Molecular weight
PI: Isoelectric point
ML: Maximum likelihood
MeJA: Methyl jasmonate
ABA: Abscisic acid
GA: Gibberellin
IAA: indoleacetic acid
ATBP-1: AT-enriched DNA-binding protein
Chr: Chromosome
MF: Molecular function
BP: Biological process
CC: Cellular component
JA-Me: Jasmines acid-mediated
PPI: Protein‒protein interaction
DEG: differentially expressed gene
KGD: Kiwifruit Genome Database
HMM: Hidden Markov model
FPKM: Fragments Per Kilobase per Million
FDR: False Discovery Rate
