# Supplementary figures and images for: Genome-wide identification and expression pattern analysis of the kiwifruit GRAS transcription factor family in response to salt stress
Source: BMC Genomics. 2024 Jan 2;25:12. doi: 10.1186/s12864-023-09915-z (PMC10759511; doi:10.1186/s12864-023-09915-z)

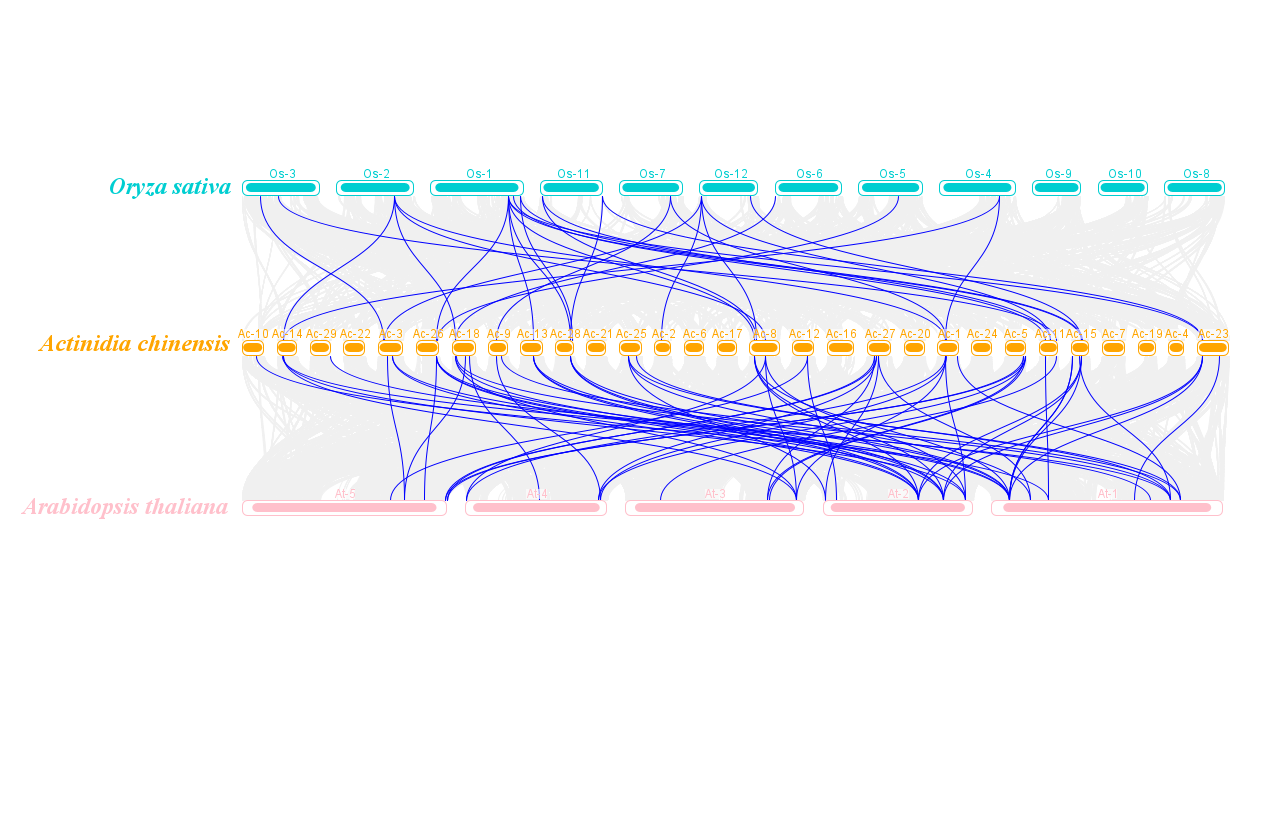

Supplement: Supplementary file 6 — Additional file 6: Additional Fig 1. Covariate distributions in kiwifruit, Arabidopsis thaliana and rice. [file 12864_2023_9915_MOESM6_ESM.png]

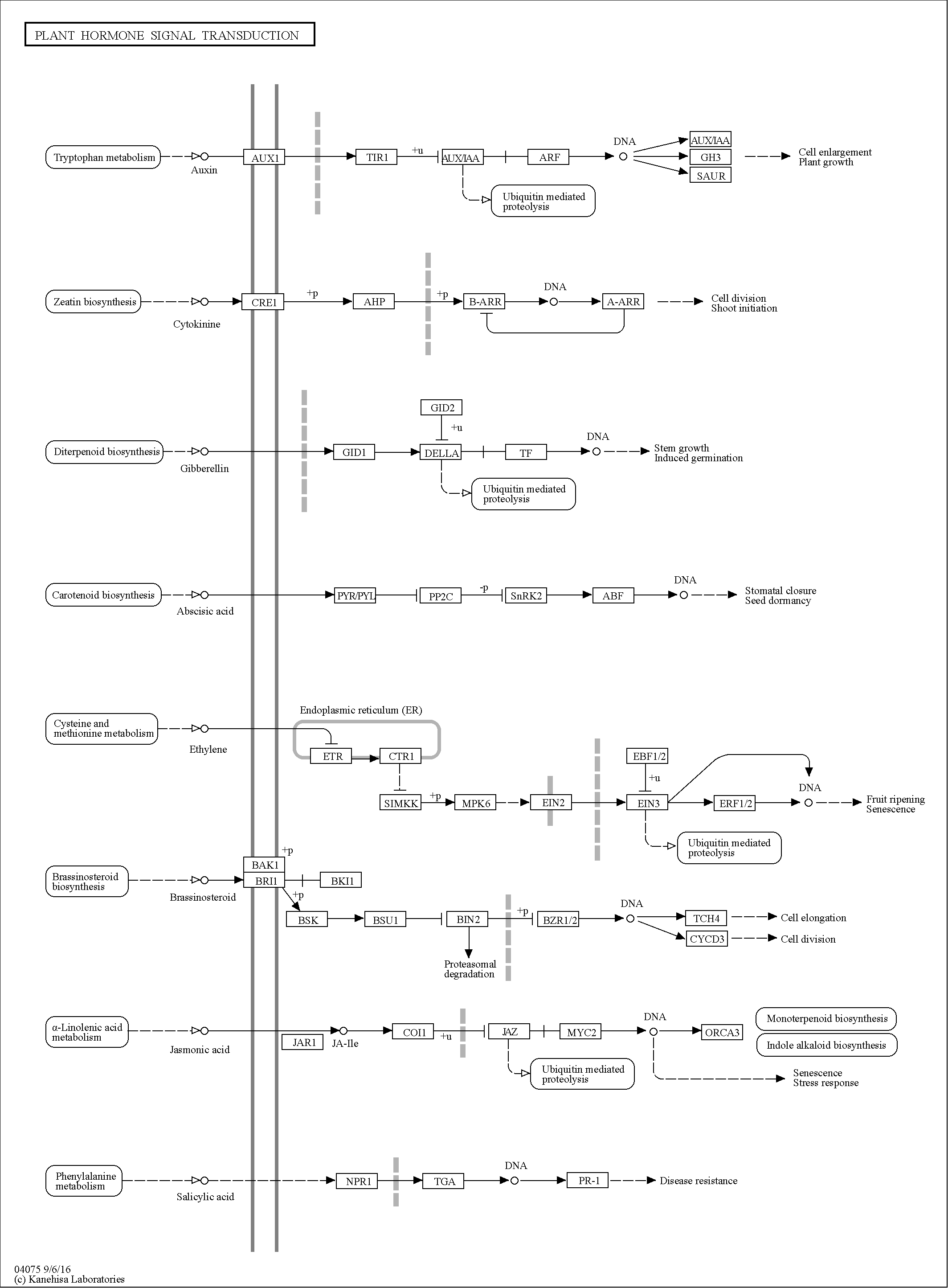

Supplement: Supplementary file 7 — Additional file 7: Additional Fig 2. AcGRAS gene KEGG is enriched to the KEGG pathway. [file 12864_2023_9915_MOESM7_ESM.png]
